# Is there a role for high dose chemotherapy and blood stem cell rescue in childhood hepatoblastoma presenting with lung metastases? A case report and literature review

**DOI:** 10.1186/1824-7288-39-65

**Published:** 2013-10-22

**Authors:** Massimo Provenzi, Francesco Saettini, Valentino Conter, Eugenia Giraldi, Carlo Foglia, Laura Cavalleri, Michele Colledan, Lorenzo D’Antiga, Giorgio Perilongo, Liviana Da Dalt

**Affiliations:** 1 Department of Pediatrics, Ospedale Papa Giovanni XXIII, Bergamo, Italy; 2 Department of Pediatrics, University of Milano-Bicocca, San Gerardo Hospital, Via Pergolesi 33, Monza, 20900, Italy; 3 Department of Surgery, Ospedale Papa Giovanni XXIII, Bergamo, Italy; 4 Department of Woman’s and Child’s Health, University Hospital of Padua, Padua, Italy

## Abstract

We report the use of high dose chemotherapy with peripheral blood stem cell rescue as a consolidation treatment for a 3-year-old child affected by metastatic hepatoblastoma, who achieved complete lung response only after conventional treatment. The patient is presently alive 27 months after high dose chemotherapy with blood stem cell rescue with no evidence of disease.

The role of high dose chemotherapy with blood stem cell rescue to consolidate the complete clearing of lung disease in metastatic hepatoblastoma remains controversial; the data available in the literature and our experience seems to suggest to keep this treatment option open to further consideration in the clinical setting of high-risk patients.

## Background

Hepatoblastoma (HB) is the most common malignant liver tumor in children. Current multidrug chemotherapy regimens and surgery allow to obtain event free survival (EFS) and overall survival (OS) rates of approximately 70-80% [1-4]. However, the outcome of children with metastatic tumor remains unsatisfactory, with EFS and OS of 25-38% and 27-62% range respectively [2-11]. Intensification of conventional chemotherapy, notably with cisplatin (CDDP), and the use of innovative combinations of drugs in a “window setting”, and more precisely, irinotecan and vincristine, are the two strategies adopted respectively by the European and North American cooperative study groups on HB to improve the present treatment outcome on these latter patients [2,12].

In the literature the use of high dose chemotherapy (HDCT) with blood stem cell (BSC) rescue has been reported in children affected by metastatic HB in 21 cases. This report aiming to review the pertinent literature on this subject and our personal observation may serve to provide a more comprehensive understanding of the role of this therapeutic approach in children with HB and high-risk features of treatment failure, notably metastases, and potentially to support further stringent clinical investigation.

## Case presentation

A three-year-old boy was referred to our center because of an asymptomatic abdominal mass. At the time of diagnosis, the alpha-feto protein (AFP) serum level was 192,269 ng/mL (normal value < 10 ng/mL) and the platelet count 486,000/mcl. The abdominal computed tomography (CT) showed an intrinsic hepatic tumor mass involving extensively the right hepatic lobe (segment VI, VII and VIII). The mass was classified as PRETEXT II (tumor volume: 13×12×10 cm) [13]. The surgical biopsy was in favor of an epithelial HB. The chest CT scan showed bilateral widespread metastatic lung disease. After obtaining informed consent, the patient was treated according to SIOPEL 4 protocol with CDDP 70 mg/sqm as a 24-hour continuous infusion (CI) on day 1, 7, and 14 (first dose of the first block CDDP at 80 mg/sqm as a 24-hour CI) and doxorubicin (DOXO) 30 mg/sqm as a 48-hour CI on day 8 and 9; this block had to be repeated three times (omitting last CDDP) at one week interval. The abdominal CT scan performed thereafter exhibited a reduction of the extension of the hepatic mass (7.5×5.1×4.5 cm), now involving only segment VII and VIII, while the chest CT scan showed only a partial response (PR) of the lung lesions, which remained bilateral and widespread. The AFP level decreased to 588 ng/ml. The surgical resection of the lung lesions was not performed because of the multifocality and the bilaterality of the disease. The patient underwent right hepatectomy and the histology was refined as a fetal HB with a rich macrotrabecular component (80% of the mass). The microscopic margins were clear. The AFP value further declined to 44.3 ng/ml 13 days after surgery. Post-surgery chemotherapy consisted of carboplatin (CARBO) 500 mg/sqm on day 1, 22 and 43, and DOXO 20 mg/sqm as a 24-hour CI on day 1, 2, 22, 23, 43, and 44. At the end of the treatment the AFP returned to normal (7.7 ng/ml) and the chest CT scan revealed no signs of residual lung disease.

Fearing that the late response of the metastases (i.e. at least a partial response to preoperative chemotherapy and complete remission of pulmonary metastases only after postoperative chemotherapy, regardless AFP levels) could be a dismal prognostic factor it was elected, with parental agreement, to complete therapy with a course of HDCT and peripheral BSC rescue. Autologous BSC were harvested in 2 days (total 1.3 × 10^6^ and 3 × 10^6^ CD34+ cells/kg), after G-CSF stimulation. As used in our center for other metastatic solid tumors, the conditioning regimen consisted of CARBO 500 mg/sqm/die from day -5 to day -3; etoposide 300 mg/sqm/die from day -5 to day -3; melphalan 120 mg/sqm/die on day -2. On day 0 and day +1 BSC (3 × 10^6^ and 1.3 × 10^6^ CD34+ cells/kg) were infused. From day +4 to day +14 G-CSF was given; neutrophil count was over 500/mm^3^ since day +12 while platelet engraftment (>20000/mcl, transfusion independent) was documented from day +13. Toxicity was modest and represented by mucositis and fever of unknown origin. Presently the patient remains disease-free, with normal AFP levels and no radiological evidence of disease, 27 months following HDCT with peripheral BSC rescue.

## Summary of the literature

The use of HDCT with BSC rescue has been used in a number of patients with HB at the time of tumor recurrence [10,14-18], but in only 22 of them, including the one here reported, as a first line treatment in patients with metastatic disease [14,15,19,20]. These 22 patients represent the focus of this report. In 2 of them a double autologous BSC rescue instead of conventional post-operative chemotherapy was performed; one of these underwent BSC rescue with persisting lung involvement. Both patients relapsed, but interestingly one of them was then rescued with irinotecan alone [14]. Fourteen patients did not achieve PR of the target lesions after conventional pre-operative chemotherapy (POC) and without further specifications regarding the tumor status at the time of HDCT all were then treated with HDCT with BSC rescue. Of these patients 2 died of toxicity of the procedure and only 4 were reported alive with no evidence of disease [20].

The last subgroup of children of whom we have detailed information encompasses 6 patients who achieved at least a PR after POC, and after conventional partial hepatectomy and post-operative chemotherapy underwent HDCT with BSC rescue, while in complete remission (CR) of lung metastases (see Table 1).

**Table 1:** High dose chemotherapy with blood stem cell rescue following post-operative chemotherapy for metastatic HB patients achieving partial response after pre-operative chemotherapy

**Reference and number of patients**	**Age/Sex**	**PRETEXT**	**Previous regimen**	**Tumor status at the time of HDCT**	**Conditioning regimen**	**BSC source**	**Outcome**
Matsunaga T [19]: 4 patients	Unknown	Any PRETEXT M+	CDDP, THP-ADR	AFP↑	CARBO, VP16, MLP	PB	3 NED 16 mts after BSC rescue 1 DoD
Nishimura SI [15]: 1 patient	10 yr/M	IV M+	CDDP, DOXO	NED	DOXO, VP16, CARBO, 5-FU	BM	NED 6 yr after BSC rescue
Provenzi M 2013: 1 patient	3 yr/M	II M+	CDDP, DOXO	NED	CARBO, VP16, MLP	PB	NED 27 mts after BSC rescue

*Abbreviations:BSC* blood stem cell, *M +* lung metastases, *CDDP* cisplatin, *THP-ADR* THP-adriamicin, *AFP* alpha-feto protein, *CARBO* carboplatin, *VP16* etoposide, *MLP* melphalan, *PB* peripheral blood, *NED* no evidence of disease, *mts* months, *DoD* died of disease, *yr* years, *DOXO* doxorubicin, *5-FU* 5 fluorouracil, *BM* bone marrow.

In these group of children four of them obtained complete clearance of metastases with POC but presented with persisting high serum levels of AFP after post-operative chemotherapy; three of these four patients at the time of the report were alive and disease-free 16 months after BSC rescue, with normal AFP levels, while one displayed continuously increasing AFP and ultimately died of brain metastases [19]. One case presenting with multiple metastatic disease that completely responded to POC is reported alive and disease-free six years from stopping therapy [15]. Our patient was a late responder to conventional chemotherapy and, as described, is alive with no evidence of disease 27 months after BSC rescue. Thus as overall five of these 6 patients had been reported to be alive with no evidence of disease with a minimum follow-up of 16 months after BSC rescue.

This small series of twenty-two patients cannot provide a comprehensive view of the role of HDCT with BSC rescue in the treatment of HB. However, the fact that 5 out of the 6 children achieved at least a PR after conventional POC and received HDCT with autologous BSC rescue while in CR of lung metastases at the end of conventional treatment seems to indicate a possible role of this treatment modality in the management of these patients.

## Conclusions

The experiences here reported may suggest thus that HDCT with autologous BSC rescue can be of benefit for patients with metastatic HB who achieved at least a PR after POC, as a consolidation of a CR of lung metastases otherwise achieved at the end of treatment. Moreover HDCT and BSC rescue can be feasible in patients who have already received intensive conventional chemotherapy but the underlying possible severe toxicity of this modality should be clearly kept in mind and parents should be closely involved in the decision process. Of course nobody knows if in these children the previous treatment, which allowed them to achieve the complete tumor response, would have been enough for the cure. However, considering that late response to chemotherapy is indeed a matter of concern in all tumor types, to have presented the series herewith reported could serve to keep the treatment option of using HDCT with BSC rescue of children affected by metastatic HB open to further clinical investigation.

## Consent

Written informed consent was obtained from the parents of the patients for publication of this Case report. A copy of the written consent is available for review by the Editor-in-Chief of this journal.

## Abbreviations

AFP: Alpha-feto protein; BSC: Blood stem cell; CARBO: Carboplatin; CDDP: Cisplatin; CI: Continuous infusion; CT: Computed tomography; CR: Complete remission; DOXO: Doxorubicin; EFS: Event free survival; HB: Hepatoblastoma; HDCT: High dose chemotherapy; OS: Overall survival; POC: Pre-operative chemtherapy; PR: Partial response.

## Competing interests

The authors declare they have no competing interests.

## Authors’ contributions

MP, FS, VC and GP conceived the study, drafted the manuscript and evaluated all the children. EG, CF, CF and MC contributed to data acquisition and drafting of the article. LDA and LDD contributed to critical revision of the article and to final approval of the version to be published. All authors read and approved the final manuscript.
